# Changes in Home Care Clients’ Sensory Impairment Status and Its Association With Functioning Over 18 Months: A Longitudinal Register-Based Study

**DOI:** 10.1177/08982643251344053

**Published:** 2025-05-16

**Authors:** Tiina Pesonen, Heidi Siira, Visa Väisänen, Johanna Edgren, Mari Aaltonen, Sinikka Lotvonen, Satu Elo

**Affiliations:** 1 Research Unit of Health Sciences and Technology, 6370 University of Oulu, Oulu, Finland; 2 3837 Finnish Institute for Health and Welfare, Helsinki, Finland; 3 Faculty of Social Sciences and Business Studies, Department of Health and Social Management, University of Eastern Finland, Kuopio, Finland; 4 6369 Oulu University of Applied Sciences

**Keywords:** sensory impairment, home care, aging in place, functioning

## Abstract

We investigated the changes in home care clients’ vision and hearing over 18 months and examined the role of sensory impairments in maintaining their functioning. We used data from the Finnish Resident Assessment Instrument (RAI) database (*n* = 7013). Sensory impairment status was categorized by type (single or dual) and severity (mild or moderate/severe). The association between sensory impairment and functioning over 18 months was examined using binary logistic regression analysis with generalized estimating equations. Of 7013 home care clients, 48% had sensory impairment at baseline. Over 18 months, sensory impairment improved in 7% (*n* = 482) and worsened in 23% (*n* = 1605) of the clients. Sensory impairments were associated with impaired physical, cognitive, and psychosocial functioning at baseline. Especially moderate to severe dual impairment was associated with increased impairment in cognitive and physical functioning over time. Sensory impairments should be considered as an integral part of maintaining home care clients’ overall health and well-being.

## Introduction

Sensory impairments can threaten active aging, functional independence, and safe living at home of community-dwelling older people (Raina et al., 2004), and therefore, they play a central role in coping and functioning at home. In Finland, and across Europe, home care is the primary option for older adults who need regular care and support (Ministry of Social Affairs and Health, 2020) as care policy has shifted from institutional care to home care and aging in place. In addition, with the rapidly aging population, especially in Finland, the number of home care clients has increased and is likely to continue increasing in the future (Mielikäinen & Kuronen, 2019). As such, it is important to investigate changes in the sensory status of home care clients over time and to examine the role of sensory impairments in maintaining their functioning.

Vision and hearing impairments are common among older people, and their prevalence increases with age. The primary cause of hearing impairment in older adults is age-related hearing loss (Haile et al., 2021), often resulting from damage to the sensory cells. While this condition is permanent and untreatable, most cases can be managed with hearing aids. The prevalence of self-reported hearing impairment among the European population is estimated at 10% (The European Coalition on Hearing Loss and Disability, 2017). This prevalence increases to 20–30% by the age of 70 years and to 45–55% by the age of 80 years (Roth et al., 2011).

In contrast, among the Western European population, the overall prevalence of vision impairment is 7.4%, rising to 18% among 70–79 year olds and up to 36% among those aged 80–89 (Vision Loss Expert Group, 2020). However, vision impairment is not an inevitable consequence of aging. World Health Organization (WHO) estimated that globally, nearly half of vision impairment cases could potentially have been prevented or treated (WHO, 2019). Based on previous research, the main cause of moderate and severe vision impairment in Europe is uncorrected refractive errors (Burton et al., 2021), which can be solved with updated glasses, contact lenses, or surgery. According to a French study, among individuals aged 78 or older, 49% of those examined at home had uncorrected refractive errors (Naël et al., 2019). Another common cause of vision impairment in older adults is cataract, which can be treated with surgery. However, vision impairment can also be permanent, resulting from damage to the eye or visual system. For example, age-related macular degeneration (AMD), diabetic retinopathy, and glaucoma can cause permanent vision impairment if untreated. Of these, only the dry form of AMD has no available treatment. For the other conditions, early recognition and treatment are crucial to prevent permanent vision impairment. In this study, vision and hearing impairment include both permanent and treatable conditions. The prevalence of dual sensory impairment, involving both vision and hearing, among older people (≥65 years) varies across studies from 1% to 34% (Bright et al., 2023). Variation between studies is likely due to different cut-off points of impairments or whether only permanent impairments are included. Bright and colleagues (2023) identified 75 definitions of dual sensory impairment in their scoping review.

**Figure 1.**
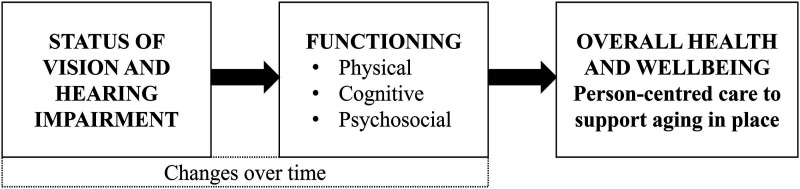
The theoretical framework of the study combines WHO’s intrinsic capacity with the WHO’s International Classification of Functioning, Disability, and Health (ICF) model.

Vision and hearing are dominant senses and have been recognized as key elements for healthy aging. The WHO introduced the concept of intrinsic capacity in 2015 as a framework for healthy aging (WHO, 2015). Intrinsic capacity is defined as the combination of an individual’s physical, cognitive, and psychological capacities (WHO, 2017). It consists of five elements: (1) locomotion, (2) vitality, (3) psychological, (4) sensory (vision and hearing), and (5) cognition. Intrinsic capacity interacts with the environment where an individual lives. This interaction together with the resources the individual can utilize determines the individual’s functional ability (WHO, 2017). Therefore, by promoting intrinsic capacity, it is possible to support the functioning of an individual. The concept of intrinsic capacity has similarities with the WHO’s International Classification of Functioning, Disability, and Health (ICF) model, which is a biopsychosocial model of functioning, disability, and health. The ICF describes functioning as a comprehensive phenomenon including the physical, psychological, and social elements of an individual’s life activities and environment (WHO, 2001). The theoretical framework of the study is illustrated in Figure 1.

Based on previous studies, sensory impairments have a broad influence on the functioning of older adults, with dual sensory impairment having more negative effects on functioning than single sensory impairment (Fuller-Thomson et al., 2022; Keller et al., 1999; Kong et al., 2023). Hearing and vision impairment, both individually and combined, are associated with declines in physical functioning among community-dwelling older adults (Kong et al., 2023; Pesonen et al., 2024). Worsening sensory impairment and a decline in physical competence in daily activities (ADL and IADL) often coincide (Jang et al., 2023), and both sensory impairments are also associated with cognitive impairment (Fuller-Thomson et al., 2022; Maharani et al., 2018). Recently, the Lancet Standing Commission has identified both sensory impairments as a risk factor for dementia (Livingston et al., 2024). In addition, a previous study on nursing home residents suggests that cognitive function tends to decline more rapidly if the residents have dual sensory impairments and simultaneously lack social engagement (Yamada et al., 2016). Furthermore, sensory impairments have negative psychosocial effects, as they are associated with increased depression (Simning et al., 2019) and loneliness (Hajek & König, 2020; Mick et al., 2018) in older adults, which may be due to reduced social participation caused by sensory impairments (Shah et al., 2020; Tseng et al., 2022). A previous study found that social isolation increased more among individuals with a single hearing impairment compared to those without sensory impairment over an 8-year follow-up period (Huang et al., 2024).

To better understand the role of sensory impairments in the overall functioning, health, and well-being of home care clients, it is crucial to investigate how sensory impairments are associated with changes in various elements of functioning. We explored changes in home care clients’ sensory impairment status, including types (single vision or hearing and dual sensory impairment) and severities (mild and moderate/severe) of impairment. Furthermore, we examined the association of sensory impairment status with functioning, including physical, cognitive, and psychosocial functioning, at baseline and over 18 months. The results can be utilized in developing social and healthcare services, including implementing timely interventions that support independence and safe living at home while preventing the need for 24-hour care.

## Methods

### Data

The data in this prospective longitudinal study were retrieved from the Finnish Resident Assessment Instrument (RAI) database. The RAI is an international and standardized system for assessing the functioning and care needs of individuals (interRAI, 2021). There are two RAI instruments for home care settings: the Minimum Data Set for Home Care instrument (MDS-HC) and the newer interRAI Home Care instrument (interRAI HC). The instruments consist of more than 300 items encompassing physical, cognitive, and psychosocial characteristics of the individuals and their living environment, as well as relevant clinical and medical information. In addition, the RAI includes different outcome scales formed from the individual items. Home care nurses conduct RAI assessments together with the client twice a year, and these assessments are used to guide care planning. Both RAI Home Care instruments have shown substantial reliability in home care settings (Hirdes et al., 2008; Landi et al., 2000).

This study uses RAI assessments of home care clients conducted between October 1, 2020, and September 30, 2022, including four assessment periods: Spring 2021 (0 months as baseline), Fall 2021 (6 months), Spring 2022 (12 months), and Fall 2022 (18 months). We included all home care clients aged 65 or older for whom all four assessments were completed, whether using the MDS-HC or interRAI HC instrument. The observation time is 7 days in MDS-HC and 3 days in interRAI HC. Other potential differences in outcome scales and items between the two instruments are described in the Measurements section. The total study sample comprised 7013 home care clients. We included all four assessments in the analysis to identify trends in changes in functioning, even though our primary focus in this study was on the situation over 18 months.

### Measurements

*Vision* was determined using the vision item of RAI, where vision is classified into 0–4 categories. Category 1 represents mild impairment (e.g., difficulty reading regular text from a book or newspaper with current eyeglasses, if used), 2 indicates moderate impairment (e.g., inability to read large print, such as newspaper headlines, but still able to see objects), 3 denotes highly impaired vision (e.g., difficulty identifying objects, though the eyes still follow objects), and 4 indicates severe impairment (e.g., no vision or the ability to see only light, colors, or shapes. Eyes do not follow objects). The guidelines instruct vision assessment to be done in adequate light and with eyeglasses if used. In this study, we used a three-level classification: (1) no impairment, including category 0; (2) mild impairment, including category 1; and (3) moderate/severe impairment, including categories 2–4.

*Hearing* was determined using the hearing item of RAI. The guidelines instruct hearing assessment to be done with a hearing aid if used. Hearing was classified into four categories from 0 to 3 in the MDS-HC instrument and into five categories (0–4) in the interRAI HC instrument. Category 0 represents adequate hearing (e.g., no difficulties in normal conversation, social interactions, or while listening to television), 1 represents mild impairment (e.g., some challenges in certain situations, such as when the other person speaks quietly), 2 indicates moderate impairment (e.g., difficulty hearing normal speech), 3 represents severe impairment (e.g., difficulties in all situations, for example, all speech from the speaker sounds like mumbling), and 4 equals no hearing. In this study, we used a three-level classification: (1) no impairment, including category 0; (2) mild impairment, including category 1; and (3) moderate/severe, including categories 2–3 in MDS-HC and 2–4 in interRAI HC.

*Dual sensory impairment* variable was formed using the aforementioned items of vision and hearing impairment. Mild dual impairment included cases where a person had mild impairment in both senses. Moderate/severe dual impairment was defined as a person having moderate/severe impairment in at least one sense, together with at least mild impairment in the other sense.

Physical, cognitive, and psychosocial elements of functioning were assessed as (1) coping with daily activities (physical functioning), (2) cognitive status (cognitive functioning), (3) depressive mood (psychosocial functioning), and (4) loneliness (psychosocial functioning).

Coping in daily activities was measured using the Activities of Daily Living Hierarchy (ADL-H) and Instrumental Activities of Daily Living (IADL Difficulty Scale in MDS-HC and IADL Capacity Hierarchy Scale in interRAI HC) scales. The ADL-H scale measures performance in activities related to four functions: eating, personal hygiene, toilet use, and locomotion. In the MDS-HC, toilet use includes the subtask of transferring on and off the toilet, while in the interRAI HC, this is excluded. The scale ranges from 0 to 6, with higher scores indicating better functioning (Morris et al., 1999). Scores 0–1 indicate independence or need for supervision. In this study, we used the ADL-H score greater than 1 to indicate the presence of ADL impairment. The same threshold has been used previously (Grue et al., 2010).

The IADL Difficulty Scale in MDS-HC assesses the capacity to complete tasks related to ordinary housework, meal preparation, and phone use, while the IADL Capacity Hierarchy Scale in interRAI HC measures the capacity to complete tasks related to ordinary housework, meal preparation, managing finances, managing medications, and shopping. Both scales are scored 0–6. Scores 0–2 indicate independence or need for supervision. It was common for home care clients to require assistance with some IADL tasks. In this study, a person was considered to have IADL impairment if the score was greater than 2. The reason for selecting the cut-off of 3 was based on the observation that even among those independent in ADLs, the mean IADL score was 2.4 at baseline.

Cognitive status was measured using the Cognitive Performance Scale (CPS). The scale ranges from 0 to 6, with a score of 6 meaning comatose or not present (Morris et al., 1994). CPS measures decision-making, short-term memory, and being understood. We used a CPS score greater than 1, which indicates mild or greater impairment, to signify the presence of cognitive decline. This threshold has also been used in previous studies (Onder et al., 2012; Yamada et al., 2016).

Depressive mood was assessed using the Depression Rating Scale (DRS), which ranges from 0 to 14. A DRS score of 3 or higher has been shown to predict a clinical diagnosis of depression (Burrows et al., 2000). In this study, depressive mood was treated as a binary variable (yes/no) with DRS scores of 3 or higher indicating the presence of a depressive mood. Loneliness was measured using a single item: *“Client expresses feeling lonely”* as a binary variable (yes/no). Loneliness had a strong correlation (0.91) with social isolation, suggesting it could be used as a proxy for social isolation.

### Statistical Analyses

The sensory impairment status variable was formed based on vision and hearing variables, including four types of impairment (mutually exclusive): (1) no sensory impairments, (2) single hearing impairment, (3) single vision impairment, and (4) dual sensory impairment. Furthermore, these types of impairment were divided into two subgroups based on the severity of impairment (mild and moderate/severe).

Changes between sensory impairment types over 18 months were illustrated using a Sankey diagram with numbers and percentages. We also examined changes in the severity of sensory impairments. Based on changes in the type and severity of sensory impairment, we classified the changes in sensory impairment status as improved, unchanged, or worsened. “Improved” and “worsened” status changes refer to one or more hierarchical steps (positive or negative, respectively) in the type (no impairment, single impairment, dual impairment) or severity (no impairment, mild, moderate/severe) of sensory impairment. “Unchanged” refers to no change in either the type or severity of sensory impairment.

The association between sensory impairment status and functioning over an 18-month period was examined using binary logistic regression analysis with generalized estimating equations (GEE), utilizing an unstructured covariance matrix. GEE is a common method for longitudinal data analysis with non-independent observations, as it accounts for the within-subject correlations present in repeated measures (Ballinger, 2004). Our analysis included four points of measurement (0 months as baseline, 6 months, 12 months, and 18 months) to identify trends in changes in functioning, even though the results focus on 18 months. We used the interaction between sensory impairments and time to determine whether changes in functioning, such as cognitive impairment, IADL impairment, ADL impairment, depressive mood, and loneliness, differed between sensory impairments and no impairment over 18 months compared to baseline. We performed separate analyses for each outcome (cognitive impairment, ADL impairment, IADL impairment, depressive mood, and loneliness) and calculated odds ratios (ORs) and 95% confidence intervals (CIs) of each outcome over 18 months. In this context, 18-month change ORs refer to an association between baseline and 18 months. An OR below 1 indicates that over time, the odds of the outcome variables is reduced with sensory impairment compared to no sensory impairment, while an OR above 1 indicates that the odds of the outcome variables increased with sensory impairment compared to no impairment over time. Functioning variables were dependent variables, while time-variant sensory impairments were independent variables (no impairment as reference). The models were adjusted for age and gender, common determinants of functioning and sensory status (Burton et al., 2021; Okabe et al., 2021), and marital status, which is associated with functioning, such as cognitive impairment (Liu et al., 2019). The analyses were conducted using IBM SPSS version 29.0.2. Statistical significance was defined as *p* < .05.

## Results

### Sensory Impairment and Functioning at Baseline

Of the 7013 home care clients, 19.2% had a single hearing impairment, 15.2% had a single vision impairment, and 13.2% had a dual sensory impairment at baseline (Table 1). The majority of the sensory impairments were mild. Home care clients with hearing, vision, and dual sensory impairment (compared to no impairment) were older (mean age 85.6, 81.6, 86.6, respectively, compared to 80.6), had been home care clients for longer (3.5, 3.9, 3.7 compared to 3.4 years), and had a lower prevalence of being married or cohabiting (13.7%, 14.8%, 15.1% compared to 17.8%). The prevalence of women among those with sensory impairments was slightly lower (68.6%, 67.5%, 67.3%) than among those without sensory impairments (70.0%). Cognitive impairment was more severe (mean 1.42, 1.34, 1.51 compared to 1.22) among home care clients with hearing, vision, and dual sensory impairment (compared to no impairment). The majority of cognitive impairment was mild to moderate with all sensory impairments. The mean score of ADL was the lowest with single hearing impairment (0.56), indicating better coping with daily activities. It was followed by no impairment (0.60), dual sensory impairment (0.66), and the score was the highest with single vision impairment (0.78). Home care clients with hearing, vision, and dual sensory impairment (compared to no impairment) more often needed help in instrumental daily activities (mean 2.78, 3.05, 3.20, respectively, compared to 2.47). The prevalence of depressive mood (9.7%, 13.0%, 11.8% compared to 8.2%) and loneliness (27.7%, 29.2%, 32.0% compared to 23.0%) was higher among persons with hearing, vision, or dual sensory impairment compared to no impairment.

**Table 1.** Characteristics of the Home Care Clients (*n* = 7013) by Type of Sensory Impairment at Baseline.

	No impairment, *n* = 3680	Single hearing impairment, *n* = 1347	Single vision impairment, *n* = 1063	Dual impairment, *n* = 923
Age, mean (SD)	80.6 (7.51)	85.6 (6.79)	81.6 (7.78)	86.6 (6.75)
Years as home care client, mean (SD)	3.4 (3.79)	3.5 (3.49)	3.9 (3.74)	3.7 (3.57)
Gender, % (*n*)				
Male	30.0 (1103)	31.4 (423)	32.5 (346)	32.7 (302)
Female	70.0 (2577)	68.6 (924)	67.5 (717)	67.3 (621)
Marital status, % (*n*)				
Married or cohabiting	17.8 (654)	13.7 (184)	14.8 (157)	15.1 (139)
Single	15.7 (579)	12.4 (167)	17.0 (181)	12.0 (111)
Widowed	36.5 (1344)	45.1 (607)	38.3 (407)	44.5 (411)
Divorced or separated	14.3 (526)	9.9 (134)	12.6 (134)	8.5 (78)
N/A	15.7 (577)	18.9 (255)	17.3 (184)	19.9 (184)
Severity of sensory impairment, % (*n*)				
Mild impairment (1)	N/A	77.7 (1047)	80.2 (853)	54.3 (501)
Moderate/severe impairment (2–4)	N/A	22.3 (300)	19.8 (210)	45.7 (422)^a^
Functioning				
Cognition (CPS, 0–6)				
Mean (SD)	1.22 (1.07)	1.42 (1.02)	1.34 (1.18)	1.51 (1.13)
No impairment (0–1), % (*n*)	56.5 (2080)	45.7 (616)	53.7 (571)	45.5 (420)
Mild/moderate impairment (2–3), % (*n*)	41.7 (1533)	52.4 (706)	43.0 (457)	51.3 (473)
Severe impairment (4–6), % (*n*)	1.8 (67)	1.9 (25)	3.3 (35)	3.2 (30)
Activities of daily living (ADL-H, 0–6)				
Mean (SD)	0.60 (1.17)	0.56 (1.04)	0.78 (1.32)	0.66 (1.16)
Independent/supervision (0–1), % (*n*)	84.9 (3125)	85.4 (1150)	80.1 (851)	82.7 (763)
Mild/moderate impairment (2–3), % (*n*)	10.8 (400)	12.0 (161)	13.5 (144)	13.0 (120)
Severe impairment (4–6), % (*n*)	4.2 (155)	2.6 (36)	6.3 (68)	4.3 (40)
Instrumental activities of daily living (IADL, 0–6)				
Mean (SD)	2.47 (1.88)	2.78 (1.82)	3.05 (1.89)	3.2 (1.84)
Independent/supervision (0–2), % (*n*)	58.9 (2169)	49.7 (669)	45.1 (479)	40.1 (370)
Mild/moderate impairment (3–4), % (*n*)	21.5 (791)	27.1 (365)	25.2 (268)	29.3 (270)
Severe impairment (5–6), % (*n*)	19.6 (720)	23.2 (313)	29.8 (316)	30.7 (283)
Depressive mood (DRS ≥3), % (*n*)	8.2 (301)	9.7 (131)	13.0 (138)	11.8 (109)
Loneliness, % (*n*)	23.0 (844)	27.7 (373)	29.2 (310)	32.0 (295)

^a^Moderate to severe impairment in at least one sense.

### Changes in Sensory Impairment Status Over 18 Months

**Figure 2.**
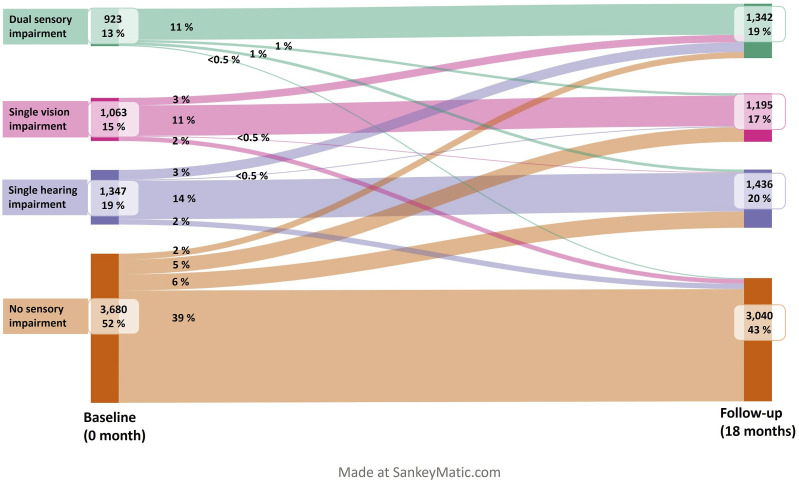
Prevalence of sensory impairments among home care clients (*n* = 7013) and changes between sensory impairment types over 18 months.

Figure 2 shows the changes in the types of sensory impairments among home care clients over an 18-month period. At baseline, 52% of the home care clients had normal vision and hearing, while after 18 months, this was true for 43% of the clients. Thus, nearly one-fifth of those who did not have a sensory impairment at the baseline had impaired vision and/or hearing after 18 months. The prevalence of single sensory impairments remained essentially the same over 18 months, while the prevalence of dual sensory impairments increased from 13% to 19%. The number of dual sensory impairments increased by 45% from 923 to 1342, after 18 months.

Although hearing and vision status worsened between sensory status groups in most cases (no impairment changed to single or dual impairment, or single impairment changed to dual impairment), some clients experienced improvements in their senses. One-tenth of those who had a single sensory impairment at the baseline had no sensory impairment after 18 months. Additionally, for those who had a dual sensory impairment at the beginning, 3% had no sensory impairment after 18 months.

In addition to changes between sensory impairment types, there were changes in the severity of the impairments. When considering the changes across types and severity over 18 months, sensory impairment status improved in 7% (*n* = 482), remained the same in 70% (*n* = 4888), and worsened in 23% (*n* = 1605) of the home care clients. In 38 cases (<1%), sensory status changed from one single sensory impairment to another single sensory impairment over 18 months.

### Time-Variant Sensory Impairment Associated With Functioning Over 18 Months

Table 2 shows that all sensory impairments (hearing, vision, and dual) and severity of impairments (compared to no impairment), except moderate/severe single vision impairment, were statistically associated with cognitive impairment at baseline. Over 18 months, moderate/severe single vision and dual impairment (compared to no impairment) were associated with 37% and 29% higher odds of cognitive impairment (OR 1.37, 95% CI 1.10–1.72; OR 1.29, 95% CI 1.09–1.52). The changes in odds at 6 and 12 months are presented in the supplementary material (Table S1).

**Table 2.** Adjusted Baseline Odds and 18 Months Change in Odds of Cognitive Impairment, ADL Impairment, IADL Impairment, Depressive Mood, and Loneliness by Sensory Impairment Status in Independent Binary Logistic Regression Analysis With Generalized Estimating Equations.

	Baseline (0 month)		Follow-up (18 months change)	
	OR (95 % CI)	*p*-value	OR 95 % CI)	*p*-value
Cognitive impairment (CPS ≥2)				
No impairment (refr.)				
Single hearing impairment				
Mild	**1.29 (1.16–1.43)**	**<0.001**	0.98 (0.88–1.09)	0.661
Moderate/severe	**1.40 (1.17–1.69)**	**<0.001**	1.05 (0.87–1.26)	0.619
Single vision impairment				
Mild	**1.19 (1.06–1.32)**	**0.002**	1.06 (0.94–1.20)	0.328
Moderate/severe	0.97 (0.77–1.21)	0.778	**1.37 (1.10–1.72)**	**0.006**
Dual impairment				
Mild	**1.50 (1.30–1.73)**	**<0.001**	0.93 (0.80–1.08)	0.315
Moderate/severe	**1.63 (1.38–1.93)**	**<0.001**	**1.29 (1.09–1.52)**	**0.003**
IADL impairment (IADL ≥3)				
No impairment (refr.)				
Single hearing impairment				
Mild	**1.22 (1.09–1.37)**	**<0.001**	1.07 (0.93–1.22)	0.341
Moderate/severe	**1.59 (1.29–1.95)**	**<0.001**	0.94 (0.75–1.16)	0.538
Single vision impairment				
Mild	**1.40 (1.25–1.57)**	**<0.001**	0.94 (0.82–1.08)	0.384
Moderate/severe	**1.61 (1.29–2.02)**	**<0.001**	1.15 (0.89–1.50)	0.293
Dual impairment				
Mild	**1.42 (1.22–1.65)**	**<0.001**	1.00 (0.84–1.18)	0.993
Moderate/severe	**2.23 (1.88–2.64)**	**<0.001**	0.96 (0.80–1.14)	0.617
ADL impairment (ADL ≥2)				
No impairment (refr.)				
Single hearing impairment				
Mild	1.04 (0.89–1.21)	0.648	1.14 (0.97–1.35)	0.122
Moderate/severe	1.14 (0.88–1.48)	0.320	1.06 (0.80–1.41)	0.679
Single vision impairment				
Mild	**1.24 (1.06–1.44)**	**0.006**	1.15 (0.97–1.39)	0.101
Moderate/severe	**1.67 (1.23–2.27)**	**0.001**	1.00 (0.72–1.39)	0.988
Dual impairment				
Mild	1.07 (0.89–1.30)	0.464	1.20 (0.98–1.46)	0.079
Moderate/severe	**1.36 (1.07–1.72)**	**0.013**	**1.30 (1.03–1.63)**	**0.026**
Depressive mood (DRS ≥3)				
No impairment (refr.)				
Single hearing impairment				
Mild	**1.39 (1.12–1.73)**	**0.003**	0.87 (0.68–1.13)	0.299
Moderate/severe	**1.73 (1.22–2.44)**	**0.002**	0.83 (0.58–1.19)	0.309
Single vision impairment				
Mild	**1.69 (1.36–2.10)**	**<0.001**	**0.76 (0.58–0.99)**	**0.040**
Moderate/severe	**2.08 (1.41–3.06)**	**<0.001**	0.62 (0.38–1.03)	0.064
Dual impairment				
Mild	**1.90 (1.48–2.45)**	**<0.001**	0.84 (0.63–1.12)	0.232
Moderate/severe	**1.97 (1.78–2.63)**	**<0.001**	1.07 (0.79–1.47)	0.655
Loneliness				
No impairment (refr.)				
Single hearing impairment				
Mild	**1.19 (1.05–1.36)**	**0.007**	0.89 (0.77–1.03)	0.105
Moderate/severe	1.13 (0.90–1.41)	0.304	1.12 (0.88–1.42)	0.377
Single vision impairment				
Mild	**1.32 (1.15–1.50)**	**<0.001**	0.89 (0.77–1.02)	0.139
Moderate/severe	1.05 (0.79–1.39)	0.743	1.03 (0.77–1.38)	0.824
Dual impairment				
Mild	**1.44 (1.22–1.69)**	**<0.001**	0.95 (0.79–1.14)	0.598
Moderate/severe	**1.63 (1.36–1.94)**	**<0.001**	0.95 (0.79–1.14)	0.551

Adjusted with age, gender, and marital status. Bold text indicates statistical significance at *p* < .05.

All sensory impairments and severity of impairments (compared to no impairment) were associated with IADL impairment at baseline. Odds for IADL impairment were highest with moderate/severe dual impairment (OR 2.23, 95% CI 1.88–2.64) at baseline. Over 18 months, no significant differences were observed in IADL impairment by hearing, vision, or dual sensory impairment compared to no sensory impairment.

Single vision impairment both mild (OR 1.24, 95 % CI 1.06–1.44) and moderate/severe (OR 1.67, 95% CI 1.23–2.27) and moderate/severe dual impairment (OR 1.36, 95 % CI 1.07–1.72) were associated with ADL impairment at baseline. Moderate to severe dual sensory impairment was associated with higher 30% odds of ADL impairment over 18 months (OR 1.30, 95% CI 1.03–1.63).

At baseline, all sensory impairments and severity of impairments increased the odds of depressive mood (compared to no impairment), while the highest odds were with moderate/severe single vision impairment (OR 2.08, 95% CI 1.41–3.06), and moderate/severe dual impairment (OR 1.97, 95% CI 1.78–2.63). Over 18 months, mild single vision impairment was associated with 24% lower odds of depressive mood compared to no impairment (OR 0.76, 95% CI 0.58–0.99).

Mild single hearing impairment (OR 1.19, 95% CI 1.05–1.36), mild single vision impairment (OR 1.32, 95% CI 1.15–1.50), as well as dual sensory impairments of both severities (OR 1.44, 95% CI 1.22–1.69, OR 1.63, 95% CI 1.36–1.94, respectively) were statistically associated with loneliness at baseline. No statistically significant differences were observed in loneliness over time by any sensory impairment compared to no sensory impairment.

## Discussion

We examined the association between sensory impairments and functioning, as well as whether changes in functioning over 18 months differ based on the type of sensory impairment. Furthermore, we explored changes in sensory impairment status over 18 months. Our results demonstrate that sensory impairments are associated with impaired cognitive, physical, and psychosocial functioning. In addition, moderate/severe single vision impairment and dual impairment, compared to no impairment, increase the odds of cognitive impairment over time, and moderate/severe dual impairment also increases the odds of impaired physical function over time. This strongly suggests that both vision and hearing play a significant role in maintaining the functioning of older people. Efforts should be targeted toward preventing the deterioration of the sensory status of home care clients.

The results show that sensory impairments are common among home care clients and that sensory status is not permanent for all cases, as it changes over time and may even improve, perhaps if a person has undergone treatments such as cataract surgery or obtained new up-to-date glasses or hearing aids. Current information on the proportion of sensory impairments of home care clients that could be managed or treated is not available, but based on previous studies, it might be considerable. The Lancet Global Health Commission has estimated that in Western Europe over 50 % of moderate and severe vision impairments are due to uncorrected refractive errors, which are treatable with up-to-date glasses (Burton et al., 2021). In a previous Finnish study, 22% of vision impairment in older people (>75 years old) was correctable with glasses and 25% with cataract surgery (Lupsakko, 2004). In addition, the use of hearing aids among older people has been found to be low, with only one-third of people with hearing impairment using hearing aids (Reed et al., 2023). The vision and hearing of home care clients should be regularly screened to identify individuals with sensory impairments, not only because sensory status can change over time but also to ensure timely interventions, such as treatment, assistive devices, or rehabilitation.

The findings indicate that the type of sensory impairment, as well as the severity of the impairment, was associated with different elements of functioning. Our results support findings from previous studies that hearing impairment does not increase the risk of impaired IADL or ADL as much as vision or dual sensory impairment (Guthrie et al., 2018; Keller et al., 1999). Hearing impairment alone was not associated with ADL impairment at baseline, a finding consistent with a previous study (Brennan et al., 2005), which showed that vision impairment and dual sensory impairment, along with the severity of these impairments, increase the risk of difficulty in daily living activities, while hearing impairment alone does not significantly impact ADL performance.

It is important to note that, according to previous studies (Tang et al., 2023), cognition is strongly associated with ADL performance. Therefore, the cumulative effects of sensory impairments and cognitive impairment on ADL performance should be considered, as this may help explain our findings regarding cognition, which were partly inconsistent with previous studies. Our results show that cognitive impairment was more strongly associated with single hearing than with vision impairments, whereas previous studies have reported contrasting findings (Fischer et al., 2016; Fuller-Thomson et al., 2022). As ADL is a key indicator of the ability to live independently at home (Vossius et al., 2019), a combination of vision impairment and cognitive impairment may exacerbate ADL difficulties to the point where independent living at home becomes impossible, even with home care services. Therefore, older adults with both more severe vision impairments and impaired cognition may be underrepresented in the data sample, which could reduce the strength of the association. In contrast, since hearing impairment alone does not significantly increase ADL impairment, clients with a single hearing impairment may still be able to live at home, even with advanced cognitive impairment. Moreover, our findings support previous evidence of an association between hearing impairment and cognitive impairment (Lin et al., 2013; Livingston et al., 2024).

Although all the elements of functioning of home care clients seem to decline over time, which is natural considering the biological process of aging, sensory impairments are associated with some of them. One notable finding was that not only did clients with dual sensory impairment have the highest odds of impaired cognition, but the odds of cognitive impairment increased more over time in those with moderate/severe dual sensory impairment compared to those with no impairment. Similar findings were reported in a previous study where cognitive decline was faster when nursing home residents had dual sensory impairments and lacked social engagement (Yamada et al., 2016). In addition, the odds of ADL impairment increased more over 18 months in home care clients with moderate/severe dual impairment compared to those with no sensory impairment. The higher odds of ADL impairment over time with moderate/severe dual impairment may be linked to an increased prevalence of impaired cognition, as impaired cognition is associated with ADL difficulties (Tang et al., 2023). These findings may suggest that the risk for dementia may accumulate with dual sensory impairment, corroborated by a Lancet Standing Commission 2024 report, where both sensory impairments were identified as risk factors for dementia (Livingston et al., 2024). Thus, treating, managing, and preventing vision and hearing impairments, when possible, is crucial as it may potentially help in maintaining cognitive function and even delaying the onset of dementia.

Based on the results, all sensory impairments were associated with depressive mood and loneliness. Depressive mood was most common with single vision impairment and dual sensory impairment. However, an interesting finding was that, over 18 months, mild vision impairment was associated with 25% lower odds of depressive mood. This may indicate an adaptation to sensory impairment, where individuals have adjusted and possibly developed coping strategies. In addition, the result may be explained by the fact that the prevalence of depressive mood was already high at baseline among those with impaired vision. The Lancet Standing Commission has identified depression and social isolation as modifiable risk factors for dementia (Livingston et al., 2024). In our study, loneliness had a strong correlation with social isolation, indicating that the results for social isolation would be similar to those for loneliness. Therefore, treating, managing, and preventing sensory impairment affects cognition not only directly but also indirectly through other risk factors. In addition, as there are bidirectional associations between sensory impairment and cognitive decline (Li et al., 2023; Vu et al., 2021), it is important to target interventions aimed at preventing, treating, and managing sensory impairment for individuals with mild cognitive impairment. Consequently, we suggest that the assessment of hearing and vision should be integrated into dementia prevention programs.

Sensory impairments may not always be preventable, nor may it always be possible to treat existing impairments. However, our results underline the value of maintaining the current sensory impairment status, as the negative effects on future functioning are reduced compared to worsened sensory impairment. The finding aligns with a previous study, which showed that worsening sensory impairments increased functional disability (Jang et al., 2023). Moreover, when sensory impairments already exist, it is crucial to address them in individual care plans to ensure adequate support and motivation for an active and meaningful life despite disability. This highlights the need for nursing professionals to have expertise in restorative care and also knowledge of rehabilitation possibilities related to low vision, hearing, and dual sensory impairments. The competence of nursing professionals in these aspects can be built through continuous education together with multidisciplinary cooperation. Additionally, our results are in line with the WHO’s view that healthy aging can be achieved either by supporting intrinsic capacity or by enabling individuals with a certain level of intrinsic capacity to engage in meaningful activities (WHO, 2017).

Overall, our findings support the WHO’s framework of intrinsic capacity, which identifies vision and hearing as key elements for healthy aging (WHO, 2015). This indicates that special attention should be paid to sensory impairments, as they significantly affect older people’s overall health, both current and the rate of change in functioning, and independence. Regular screening is pivotal to recognizing sensory impairments. Fortunately, Finland is a forerunner, as home care clients are regularly screened for sensory impairments using the RAI tool. However, in addition to screening, national or regional protocols are needed to outline the next steps after impairments are identified. This should include guidance on where clients can be referred for further assessment of vision and hearing, how to ensure they can access these services even if their physical or cognitive functioning is impaired, and plans for monitoring the progression of impairments. This issue is especially important for home care clients who do not have informal caregivers. In addition, continuing education on sensory impairments is needed for home care employees.

### Limitations

The present study aimed to examine the association between sensory impairments and the elements of functioning of home care clients using comprehensive register data and a longitudinal study design. However, we acknowledge that the selected variables might not comprehensively cover the multifaceted concept of functioning. Physical functioning, for example, encompasses factors beyond coping in activities of daily living. Additionally, using both the MDS-HC and interRAI HC instruments can be seen as a limitation, as although they are very similar, they are not identical. For example, the IADL scale in interRAI HC includes more items than in MDS-HC. However, we conducted a sensitivity analysis by adding the instrument variable to the regression model, and the results remained consistent. Moreover, although the dataset was substantial in size, it may not represent all home care clients in Finland. The sample does not include those who moved to 24/7 assisted living facilities or those who died during the follow-up period. Therefore, the data may be missing the frailest home care clients, which could affect the results by reducing the strength of associations. It is also important to note that the RAI assessment is a subjective appraisal in nature, and the individual assessments may be influenced by the assessor and their expertise, potentially affecting our results. For example, it is unclear whether the assessments were conducted as outlined in the guidebook (e.g., using a text sample and ensuring adequate lighting). The results should also consider the possibility that changes in sensory impairment status may be a result of measurement error, as we lacked information on whether any individuals received treatment, new glasses, or hearing or visual aids. However, the reliability of the RAI instrument itself has been validated through previous research studies (Hirdes et al., 2008; Landi et al., 2000), and hearing and vision items have also been found to be reliable (Dalby et al., 2009).

## Conclusion

Sensory impairments are common among home care clients, and especially dual impairment appears to increase impairment in cognitive and physical functioning. As sensory impairment status can change over time, regular vision and hearing screenings are essential to identify any sensory impairments and provide timely interventions. Since sensory impairments, even when mild, are associated with different elements of functioning, it is crucial to understand the impact of vision and hearing on the independence, overall health, and well-being of home care clients. Sensory status should be further integrated into care plans to support a holistic approach. When sensory impairments cannot be improved, maintaining the current sensory status becomes pivotal, as further decline could increase the negative effects on functioning. By prioritizing the prevention and management of sensory impairments, home care can empower clients to preserve their independence, enhance their quality of life, and potentially delay or prevent further decline in physical, cognitive, and psychosocial functioning.

## Supplemental Material

Supplemental Material - Changes in Home Care Clients’ Sensory Impairment Status and Its Association With Functioning Over 18 Months - A Longitudinal Register-Based Study Supplemental Material for Changes in Home Care Clients’ Sensory Impairment Status and Its Association With Functioning Over 18 Months - A Longitudinal Register-Based Study by Tiina Pesonen, Heidi Siira, Visa Väisänen, Johanna Edgren, Mari Aaltonen, Sinikka Lotvonen, and Satu Elo in Journal of Aging and Health

## Data Availability

The data that support the findings of this study are available from the corresponding author upon reasonable request.*
