# Supplementary material for: Changes in Home Care Clients’ Sensory Impairment Status and Its Association With Functioning Over 18 Months: A Longitudinal Register-Based Study
Source: J Aging Health. 2025 May 16;38(5-6):325–36. doi: 10.1177/08982643251344053 (PMC13103332; doi:10.1177/08982643251344053)
Supplement: Supplemental Material - Changes in Home Care Clients’ Sensory Impairment Status and Its Association With Functioning Over 18 Months - A Longitudinal Register-Based Study [file sj-pdf-1-jah-10.1177_08982643251344053.pdf]

APPENDIX

Table S1. Adjusted 6 and 12 months change in odds of cognitive impairment, ADL impairment, IADL impairment, depressive mood, and loneliness by sensory impairment status in independent binary logistic regression analysis with generalized estimating equations.

	**6 months change**		**12 months change**	
	**OR (95 % CI)**	**P-value**	**OR (95 % CI)**	**P-value**
**COGNITIVE IMPAIRMENT (CPS ≥ 2)**				
**No impairment (refr.)**				
**Single hearing impairment**				
Mild	1.03 (0.87-1.21)	0.754	0.99 (0.92-1.06)	0.777
Moderate/severe	1.03 (0.90-1.18)	0.622	1.03 (0.87-1.21)	0.754
**Single vision impairment**				
Mild	1.02 (0.94-1.10)	0.704	1.03 (0.93-1.14)	0.535
Moderate/severe	**1.22 (1.05-1.42)**	**0.009**	**1.27 (1.03-1.56)**	**0.025**
**Dual Impairment**				
Mild	1.03 (0.94-1.14)	0.518	0.96 (0.84-1.09)	0.508
Moderate/severe	1.00 (0.91-1.10)	0.925	**1.17 (1.01-1.35)**	**0.032**

Table S1. Adjusted 6 and 12 months change in odds of cognitive impairment, ADL impairment, IADL impairment, depressive mood, and loneliness by sensory impairment status in independent binary logistic regression analysis with generalized estimating equations.

**IADL IMPAIRMENT (IADL ≥ 3)**				
**No impairment (refr.)**				
**Single hearing impairment**				
Mild	0.96 (0.89-1.04)	0.362	0.98 (0.88-1.10)	0.795
Moderate/severe	0.94 (0.82-1.08)	0.360	1.00 (0.83-1.20)	0.984
**Single vision impairment**				
Mild	0.92 (0.84-1.01)	0.068	**0.87 (0.78-0.98)**	**0.026**
Moderate/severe	0.99 (0.84-1.16)	0.898	1.08 (0.86-1.35)	0.534
**Dual Impairment**				
Mild	0.94 (0.83-1.05)	0.269	0.94 (0.81-1.09)	0.426
Moderate/severe	0.94 (0.84-1.05)	0.289	0.94 (0.81-1.09)	0.431
**ADL IMPAIRMENT (ADL ≥ 2)**				

Table S1. Adjusted 6 and 12 months change in odds of cognitive impairment, ADL impairment, IADL impairment, depressive mood, and loneliness by sensory impairment status in independent binary logistic regression analysis with generalized estimating equations.

**No impairment (refr.)**				
**Single hearing impairment**				
Mild	1.03 (0.92-1.15)	0.595	1.00 (0.86-1.16)	0.988
Moderate/severe	1.01 (0.83-1.23)	0.893	0.92 (0.71-1.20)	0.557
**Single vision impairment**				
Mild	1.00 (0.89-1.12)	0.937	1.07 (0.93-1.24)	0.338
Moderate/severe	0.94 (0.74-1.20)	0.629	0.97 (0.74-1.28)	0.823
**Dual Impairment**				
Mild	1.04 (0.89-1.21)	0.625	1.10 (0.92-1.32)	0.285
Moderate/severe	1.00 (0.85-1.18)	0.997	1.01 (0.89-1.37)	0.371
**DEPRESSIVE MOOD (DRS ≥ 3)**				
**No impairment (refr.)**				
**Single hearing impairment**				
Mild	0.91 (0.76-1.11)	0.355	0.89 (0.71-1.23)	0.343
Moderate/severe	0.88 (0.68-1.16)	0.366	0.99 (0.71-1.40)	0.970

Table S1. Adjusted 6 and 12 months change in odds of cognitive impairment, ADL impairment, IADL impairment, depressive mood, and loneliness by sensory impairment status in independent binary logistic regression analysis with generalized estimating equations.

**Single vision impairment**				
Mild	0.93 (0.76-1.14)	0.479	0.95 (0.75-1.22)	0.689
Moderate/severe	1.00 (0.71-1.40)	0.991	0.80 (0.52-1.24)	0.332
**Dual Impairment**				
Mild	0.90 (0.70-1-16)	0.413	0.83 (0.62-1.09)	0.171
Moderate/severe	1.04 (0.80-1.35)	0.765	1.22 (0.90-1.64)	0.198
**LONELINESS**				
**No impairment (refr.)**				
**Single hearing impairment**				
Mild	0.93 (0.84-1.03)	0.155	0.94 (0.83-1.07)	0.337
Moderate/severe	1.20 (1.00-1.44)	0.051	1.21 (0.97-1.50)	0.090
**Single vision impairment**				
Mild	0.99 (0.89-1.09)	0.784	0.94 (0.82-1.08)	0.371
Moderate/severe	1.15 (0.94-1.40)	0.182	1.11 (0.85-1.45)	0.428
**Dual Impairment**				
Mild	1.07 (0.94-1.22)	0.320	1.01 (0.86-1.19)	0.896

Table S1. Adjusted 6 and 12 months change in odds of cognitive impairment, ADL impairment, IADL impairment, depressive mood, and loneliness by sensory impairment status in independent binary logistic regression analysis with generalized estimating equations.

Moderate/severe	0.92 (0.80-1.06)	0.250	1.00 (0.85-1.17)	0.962

Adjusted with age, gender, and marital status. Bold text indicates statistical significance at $p < 0.05$.
